# One year snapshot: antipsychotic use in institute of forensic psychiatry of kosovo

**DOI:** 10.1192/j.eurpsy.2023.1136

**Published:** 2023-07-19

**Authors:** G. Halilaj, M. Gjocaj, N. Dakaj, F. Drevinja, S. Rakaj, N. Fanaj

**Affiliations:** 1 UBT Prishtinë Kosovo; 2 Institute of Forensic Psychiatry, Prishtina; 3 Alma Mater Europaea Campus College Rezonanca, Prishtina, Prishtinë; 4 QDT BIOING, Prizren, Kosovo

## Abstract

**Introduction:**

The use of antipsychotics in the treatment of the mentally ill represents a complex modality, especially in specialized institutions such as the Institute of Forensic Psychiatry of Kosovo. Current best practices are summarized in clinical guidelines, which nevertheless recognize the importance of individualizing treatment. In literature there is the scarcity of knowledge on the effectiveness of pharmacological treatment within a forensic psychiatric population.

**Objectives:**

To understand the features of the use of antipsychotics in IPFK as a prerequisite for increasing the quality and adequate treatment in psychiatric institutions in Kosovo.

**Methods:**

It is a retrospective study. The files of 100 patients admitted to IPFK for evaluation and treatment in 2021 were analysed. The age ranged from 18 to 71 years (Mage=36.33; SD=12.66). General demographic data, types of antipsychotics, their doses, their combinations were looked at. Data analysis was processed with SPSS 26 and Microsoft Excel 2016.

**Results:**

43% of patients were not prescribed any antipsychotic drugs. 38% used one antipsychotic, 16% used 2 antipsychotics at the same time and 3% used three types of antipsychotics. 12% of patients used four types of psychotropic drugs (antipsychotic, anxiolytic and mood stabilizer), 12% were on three types of psychotropic drugs, 42% did not use any type of medication. No side effects were noted. Only one case of refusal of therapy was recorded. The doses of the drugs used are within the recommended therapeutic limits. Most of the antipsychotics used were of the second generation. In 44% of cases they received Risperidone, in 17% of cases Haloperidol, in 14% of cases Olanzapine, in 5% of cases Clozapine and in only 1% Aripiprazole.

**Conclusions:**

Antipsychotic medication is the main method of treatment in IPFK, based on the specifics of the cases. The impression of overuse of several antipsychotics at the same time requires deeper professional consideration in order to avoid chemical restraint as a management method.

**Disclosure of Interest:**

None Declared

